# Predicting malaria in sudanese pregnant women: reliability of complete blood count

**DOI:** 10.1590/1806-9282.20250022

**Published:** 2025-10-17

**Authors:** Mustafa Cengiz Dura, Berk Gürsoy, Hilal Aktürk, Özgür Aslan, Metehan Hergüner, Elif Ezirmik

**Affiliations:** 1 University of Health Sciences Turkey, Bakirkoy Dr. Sadi Konuk Training and Research Hospital, Department of Obstetrics and Gynecology – Istanbul, Turkey.; 2 Kahta State Hospital, Department of Obstetrics and Gynecology – Adıyaman, Turkey.; 3 Mus State Hospital, Department of Obstetrics and Gynecology – Muş, Turkey.; 4 Karasu District Health Directorate, Department of Family Medicine – Karasu, Turkey.

**Keywords:** Malaria, Pregnancy, Platelet count, Blood platelets, Hematology, Biomarker

## Abstract

**BACKGROUND:**

Malaria remains a major public health concern in tropical regions, especially among pregnant women. It increases the risk of maternal anemia, low birth weight, preterm delivery, and infant mortality.

**OBJECTIVE:**

The aim of this study was to assess the hematological effects of malaria during pregnancy and evaluate the diagnostic value of blood count parameters, particularly platelet distribution width, as a potential biomarker.

**METHODS:**

In this retrospective study, 174 pregnant women were included: 78 with confirmed malaria and 96 malaria-negative controls. Hematological parameters such as hemoglobin, white blood cell count, platelet count, platelet distribution width, and mean platelet volume were compared between the groups. Statistical analyses included univariate and multivariate logistic regression and receiver operating characteristic curve analysis.

**RESULTS:**

There were no significant differences between the groups in terms of age, gestational week, gravida, parity, abortion, white blood cell, or mean platelet volume. However, body mass index, hemoglobin, platelet, and platelet distribution width were significantly lower in the malaria group (p<0.05). While all the four parameters predicted malaria status in univariate analysis, only platelet distribution width remained significant in multivariate analysis. Platelet distribution width demonstrated the highest diagnostic accuracy (area under the curve=0.857).

**CONCLUSION:**

Platelet distribution width may serve as a reliable and accessible hematological marker for malaria in pregnancy, particularly in resource-limited settings. Routine evaluation of complete blood count parameters could support early diagnosis and improve maternal–fetal outcomes.

## INTRODUCTION

Malaria, a parasitic disease transmitted through the bite of infected *Anopheles* mosquitoes, remains a significant global health challenge, particularly in tropical and subtropical regions^1^. In Sudan, malaria poses serious health risks, particularly for pregnant women and children under five. Each year, almost 25 million pregnant women residing in malaria-endemic regions are affected by malaria infection during childbirth^2^. Pregnant women are at risk of death due to malaria complications, which can negatively impact economic productivity and individuals’ annual income^3,4^. The implications of malaria during pregnancy are profound, with the potential to cause severe health complications for both the mother and the fetus. These complications can range from increased maternal anemia, low birth weight, and preterm delivery, to increased infant mortality^5,6^.

The intricate interplay between malaria infection and the physiological changes during pregnancy exacerbates the risk of adverse outcomes. Hematological alterations, a hallmark of malaria infection, can be particularly detrimental during gestation, affecting both maternal and fetal well-being^7,8^. A comprehensive hemogram, which includes the assessment of red blood cells (RBCs), white blood cells (WBCs), hemoglobin (Hb) levels, hematocrit (HCT), and platelets (PLTs), serves as a critical indicator of the body's response to infection and overall health status^9^.

Recent international studies from sub-Saharan Africa and Latin America have similarly reported hematological disruptions in pregnant women with malaria, highlighting the global relevance of this issue and supporting the need for region-specific investigations^10–12^.

Hemogram parameters play a pivotal role in the diagnosis and monitoring of malaria infection. The quantification of RBCs and Hb levels can reveal the presence of anemia, a common complication of malaria^13,14^. Elevated WBC count may indicate an immune response to infection, while changes in PLT count can signal the potential for coagulation issues^12^. These parameters, when analyzed collectively, can provide valuable insights into the severity of the infection and the body's response to it^13^.

This study aimed to elucidate the hematological impact of malaria infection on pregnant women by comparing the hemogram values of malaria-positive pregnant women with those of malaria-negative counterparts. By conducting a cross-sectional analysis, we seek to identify the specific hematological parameters that are most affected by malaria during pregnancy. The findings from this comparative study will not only enhance our understanding of the pathophysiological mechanisms underpinning malaria's impact on pregnancy but also inform clinical practices and interventions aimed at improving maternal–fetal health outcomes in malaria-endemic regions. Furthermore, this research endeavors to contribute to the broader discourse on maternal health in the context of infectious diseases, highlighting the need for targeted healthcare strategies that address the unique challenges faced by pregnant women in malaria-affected areas. Through this detailed examination of hemogram values, we anticipate shedding light on potential biomarkers for malaria-related complications in pregnancy, thereby facilitating early diagnosis and timely intervention.

## METHODS

This retrospective study was conducted at the Turkish Nyala Sudan Training and Research Hospital between June 2020 and February 2021 and received ethics committee approval (REF 2020/21/786). Informed consent was submitted by all subjects when they were enrolled.

A total of 185 pregnant women were included in the study. Among them, five participants in the study group and six participants in the control group were excluded from the study because their hospital records could not be accessed. Of the remaining participants, 78 pregnant women with a diagnosis of malaria infection and 96 pregnant women with aparasitemia were included in the control group. Demographic information of the participants was recorded. The hemogram parameters of the participants were examined retrospectively through hospital records.

The study enrolled pregnant women over the age of 18 who did not have any previous diagnosis of hematologic disorders. The exclusion criteria included pregnant women with pre-existing comorbidities such as hematologic disorders, liver and splenic diseases, and those who had previously been identified with a different cause of anemia. These criteria were implemented to minimize potential confounding factors and selection bias, ensuring that the study focused specifically on the hematological effects of active malaria infection during the current pregnancy.

A complete blood count was performed on an automatic machine (Sysmex XE 5000) by taking a blood sample of 4 mL of whole blood in a tube containing ethylenediaminetetraacetic acid (EDTA) from each participant after venipuncture. Blood samples were collected during routine antenatal visits by trained phlebotomists using standard aseptic techniques. All hematological analyses were conducted within 2 h of collection in the hospital's central laboratory by qualified laboratory technicians.

The instances involved pregnant individuals exhibiting symptoms and indications of uncomplicated malaria, who were subsequently proven to be infected with either *Plasmodium falciparum* or *Plasmodium vivax* by a fast diagnostic test and microscopic examination of blood smears.

The levels of Hb (g/dL), WBC (×10^3^/mL), PLT (×10^6^/mL), platelet distribution width (PDW) (%), and mean values were compared between the paracytemic study and aparacytemic control groups. Additionally, between the groups, specific PLT indices such as mean platelet volume (MPV) (fL) were also examined.

### Statistical analysis

Descriptive statistics of continuous variables were presented as mean and standard deviation, while categorical variables were summarized using frequencies (n) and percentages (%). The normality of data distribution was assessed using the Kolmogorov-Smirnov test. Group comparisons were performed using the Mann-Whitney U test and independent samples t-test as appropriate.

Univariate logistic regression analysis was initially used to assess the relationship between individual hematological parameters (body mass index [BMI], Hb, PLT, and PDW) and malaria status. Variables found to be statistically significant in univariate analysis were then included in a multivariate logistic regression model to identify independent predictors. Odds ratios (ORs), 95%CIs, and p-values were reported. Receiver operating characteristic (ROC) curve analysis was also conducted to evaluate the diagnostic performance of each parameter.

Additionally, a retrospective power analysis was performed using G*Power software, based on the PDW variable, which showed the strongest association. With an observed effect size (Cohen's d) of approximately 1.6, sample sizes of 96 (control group) and 78 (malaria group), and an alpha level of 0.05, the calculated statistical power exceeded 0.99, indicating the adequacy of the sample size and minimizing the risk of Type II error.

All statistical analyses were conducted using IBM SPSS version 25 and MedCalc version 12. A p-value <0.05 was considered statistically significant.

## RESULTS

A total of 174 patients, 96 (55.2%) in the control group and 78 (44.8%) in the malaria group, were included in the study. The demographic and blood parameter characteristics of the groups are shown in Table 1. There was no significant difference between the groups in terms of age, gestational week, gravida, parity, abortion, WBC count (×10^3^/μL), and MPV (fL) (p>0.05). On the other hand, BMI (*p<0.05), Hb (g/dL) (***p<0.001), PLT (×10^6^/μL) (***p<0.001), and PDW (%) (***p<0.001) values of the control group were significantly higher than those of the malaria group.

**Table 1:** Comparison of demographic and blood parameters between control and malaria-positive groups.

	Control group n=96 Mean (SD)	Patients with malaria n=78 Mean (SD)	p	
Age	28.45 (6.10)	28.95 (5.67)	0.579	a
BMI	26.99 (3.09)	25.87 (3.20)	*0.026	b
Gestational week	31.02 (3.47)	31.10 (3.52)	0.872	b
Gravida	2.46 (1.20)	2.65 (1.32)	0.364	b
Parity	1.07 (0.95)	1.18 (1.10)	0.649	b
Abortion	0.46 (0.71)	0.51 (0.73)	0.601	b
Hemoglobin (g/dL)	11.37 (1.60)	9.93 (1.82)	***<0.001	b
WBC (×10^3^/μL)	7.70 (2.26)	7.96 (2.37)	0.551	b
PLT (×10^6^/μL)	269.22 (76.65)	198.12 (39.74)	***<0.001	b
PDW (%)	13.76 (2.99)	9.99 (1.40)	***<0.001	b
MPV (fL)	8.97 (1.47)	8.93 (1.79)	0.694	b

a Independent samples t-test.

b Mann-Whitney U test. SD: standard deviation; BMI: body mass index; WBC: white blood cell; PLT: platelet; PDW: platelet distribution width; MPV: mean platelet volume.

* p<0.05;

*** p<0.001.

Table 2 summarizes the evaluation of parameters using both univariate and multivariate logistic regression analyses. According to the univariate model, BMI, Hb (g/dL), PLT (×10^6^/μL), and PDW (%) were found to be parameters that significantly predicted malaria status, while in the multivariate model, only PDW (%) significantly predicted malaria status.

**Table 2:** Univariate and multivariate logistic regression analysis results of each parameter.

	Univariate model			Multivariate model		
	OR	95%CI	p	OR	95%CI	P
BMI	0.891	0.807–0.984	0.023	0.928	0.815–1.056	0.256
Hemoglobin (g/dL)	0.618	0.509–0.750	<0.001	1.126	0.859–1.477	0.391
PLT (×10^6^/μL)	0.982	0.975–0.988	<0.001	0.994	0.985–1.002	0.123
PDW (%)	0.498	0.401–0.619	<0.001	0.528	0.403–0.691	<0.001

OR: odds ratio; CI: confidence interval; BMI: body mass index; PLT: platelet; PDW: platelet distribution width.

When each parameter was examined using ROC analysis, differences were observed between the groups with and without malaria for BMI, Hb (g/dL), PLT (×10^6^/μL), and PDW (%), with area under the curve (AUC) values of 0.598, 0.726, 0.767, and 0.857, respectively. Based on this finding, these parameters were found to have the ability to predict malaria (Table 3). Among these parameters, the parameter with the highest discriminatory power was found to be PDW.

**Table 3:** Reliability of hematological indices in predicting malaria.

Parameter	Malaria group	Control group	Malaria group	Sensitivity	PPV	Specificity	NPV	AUC	95%CI	p
BMI	≤26.50	38	46	58.97	54.8	60.42	64.4	0.598	0.513–0.683	0.026
	>26.50	58	32							
Hemoglobin (g/dL)	≤9.3	9	38	48.72	80.9	90.62	68.5	0.726	0.649–0.804	<0.001
	>9.3	87	40							
PLT (×10^6^/μL)	≤245	37	68	87.18	64.8	61.46	85.5	0.767	0.697–0.838	<0.001
	>245	59	10							
PDW (%)	≤12.1	32	74	94.87	69.8	66.67	94.1	0.857	0.802–0.911	<0.001
	>12.1	64	4							

AUC: area under the curve; CI: confidence interval; BMI: body mass index; PLT: platelet; PDW: platelet distribution width; PPV: positive predictive value; NPV: negative predictive value.

## DISCUSSION

The impact of malaria on hematological parameters during pregnancy is a critical area of research due to its implications for maternal and fetal health^15–17^. Our study contributes to this field by comparing blood parameters between pregnant women with and without malaria.

The absence of significant differences in age, gestational week, gravida, parity, abortion, WBC count, and MPV suggests that these factors are not markedly influenced by malaria infection. In contrast, BMI, Hb, PLT, and PDW values were significantly lower in the malaria group. Similar findings have been reported in previous studies. For instance, Agena et al. observed a significant decrease in RBC count among malaria-positive patients in Sudan^18^. The significant differences observed in BMI, Hb, PLT, and PDW are indicative of the infection's impact. Moreover, lower BMI in our malaria group may reflect increased metabolic demands or nutritional deficiencies associated with the infection. Anemia, reflected by reduced Hb levels, is a well-documented complication of malaria and poses serious risks during pregnancy^19–21^.

It is also important to consider other factors that may influence hematological parameters. Nutritional deficiencies such as iron, folate, or vitamin B12 deficiency and undiagnosed infections or inflammatory conditions may affect blood indices. Although we excluded participants with known comorbidities or other infections, the possibility of subclinical or unrecorded conditions cannot be entirely ruled out. These variables could contribute to individual variability and should be considered when interpreting the results.

The significant decrease in PLT count and PDW in the malaria group is also noteworthy. These findings support prior research indicating that thrombocytopenia is common in malaria and may increase the risk of bleeding^22–23^. One possible explanation for the lower PDW is the release of immature PLTs of similar size from the bone marrow, leading to reduced variability in PLT size^24^. The fact that PDW remained a significant predictor in both univariate and multivariate logistic regression analyses further supports its potential as a diagnostic biomarker for malaria in pregnancy^25^. This finding represents a key strength of our study, though validation in larger, prospective cohorts is necessary.

Similar reductions in PLT indices have been observed in studies from other endemic regions, including Nigeria, Ghana, and Colombia, suggesting that hematological responses to malaria in pregnancy may share consistent patterns across geographically diverse populations^10–12^.

Our study has several limitations. It was cross-sectional in nature and was conducted at a single center, which may affect the generalizability of our findings. The retrospective design limits causal inference and introduces potential issues related to data completeness. Since we relied on hospital records, we could not account for all clinical or sociodemographic variables that might have influenced the outcomes. Furthermore, as the study was conducted in an urban hospital, the findings may be subject to selection bias and may not fully reflect rural or underserved populations. Future studies should employ longitudinal, multi-center designs to track changes in hematological parameters throughout pregnancy and across diverse healthcare settings.

Additionally, the study lacked data on important confounding factors such as nutritional status, co-infections, and prenatal care variability. Although patients with known infections or comorbidities were excluded, subclinical conditions may have gone undetected. Moreover, the cross-sectional design precluded temporal analysis, thereby limiting our ability to infer causal relationships. Future longitudinal studies are needed to explore these associations over time and under varying clinical conditions.

Given its strong predictive value in our study, PDW could serve as a supportive diagnostic tool in clinical settings, particularly in resource-limited environments where advanced diagnostic methods are not always available. Including PDW in routine antenatal blood assessments may help identify pregnant women at risk for malaria-related hematological complications early in the disease course.

## CONCLUSION

Our findings support the clinical relevance of monitoring hematological parameters, particularly PDW, in pregnant women with malaria. PDW may offer a simple, cost-effective, and accessible marker for identifying malaria-related hematological disturbances, especially in resource-limited settings where diagnostic tools are scarce. Incorporating PDW evaluation into routine antenatal blood testing could aid early detection and risk stratification. However, further prospective and multi-center studies are needed to confirm these findings, establish standardized PDW thresholds, and guide evidence-based clinical application.

In parallel with hematological monitoring, strengthening intermittent preventive treatment (IPTp) programs, expanding the use of insecticide-treated nets (ITNs), and improving prenatal care services are essential components of an integrated public health strategy to reduce the burden of malaria in pregnancy.

## Data Availability

The datasets generated and/or analyzed during the current study are available from the corresponding author upon reasonable request.
